# Curcuminoids for Metabolic Syndrome: Meta-Analysis Evidences Toward Personalized Prevention and Treatment Management

**DOI:** 10.3389/fnut.2022.891339

**Published:** 2022-06-09

**Authors:** Agustina Dwi Retno Nurcahyanti, Fonny Cokro, Martha P. Wulanjati, Mona F. Mahmoud, Michael Wink, Mansour Sobeh

**Affiliations:** ^1^Department of Pharmacy, School of Medicine and Health Sciences, Atma Jaya Catholic University of Indonesia, Jakarta, Indonesia; ^2^Research Division for Natural Products Technology (BPTBA), National Research and Innovation Agency (BRIN), Yogyakarta, Indonesia; ^3^Department of Pharmacology and Toxicology, Faculty of Pharmacy, Zagazig University, Zagazig, Egypt; ^4^Institute of Pharmacy and Molecular Biotechnology (IPMB), Heidelberg University, Heidelberg, Germany; ^5^AgroBioSciences Department, Mohammed VI Polytechnic University, Ben-Guerir, Morocco

**Keywords:** metabolic syndrome, curcumin, curcuminoid, turmeric, personalized treatment, gut microbiome, meta-analysis, randomized controlled trial

## Abstract

The metabolic syndrome (MS) is a multifactorial syndrome associated with a significant economic burden and healthcare costs. MS management often requires multiple treatments (polydrug) to ameliorate conditions such as diabetes mellitus, insulin resistance, obesity, cardiovascular diseases, hypertension, and non-alcoholic fatty liver disease (NAFLD). However, various therapeutics and possible drug-drug interactions may also increase the risk of MS by altering lipid and glucose metabolism and promoting weight gain. In addition, the medications cause side effects such as nausea, flatulence, bloating, insomnia, restlessness, asthenia, palpitations, cardiac arrhythmias, dizziness, and blurred vision. Therefore, is important to identify and develop new safe and effective agents based on a multi-target approach to treat and manage MS. Natural products, such as curcumin, have multi-modalities to simultaneously target several factors involved in the development of MS. This review discusses the recent preclinical and clinical findings, and up-to-date meta-analysis from Randomized Controlled Trials regarding the effects of curcumin on MS, as well as the metabonomics and a pharma-metabolomics outlook considering curcumin metabolites, the gut microbiome, and environment for a complementary personalized prevention and treatment for MS management.

## Introduction

The metabolic syndrome (MS) is a complex health disorder and causes a substantial economic burden (1) as it involves numerous health conditions including obesity, type 2 diabetes, insulin resistance, atherosclerotic cardiovascular disease, elevated blood pressure, and dyslipidemia (2, 3). Polydrug approaches are often utilized in MS management but such therapeutics often exhibit adverse side effects, such as nausea, flatulence, and diarrhea as common side effects of metformin (4, 5). Insomnia, anxiety, asthenia, palpitations, vertigo, and blurred vision are caused by phentermine and diethylpropion (6), as well as hypoglycemia, pain on injection site, weight gain, and nausea, are frequent side effects of Neutral Protamine Hagedorn (NPH), liraglutide, and exenatide (7, 8).

Natural products offer broad pharmacological activities acting *via* multiple mechanisms on MS pathophysiological signaling (9–11). However, the lack of understanding of MS pathophysiology at the cellular and molecular level (12, 13) means that clinical reports on natural products, for instance as preventive agents, often disagree with the observed preclinical findings. Furthermore, only a few natural products and food items have been translated into clinical trials, for example, resveratrol, orange, grape, walnut, soy, and mushroom improve insulin secretion and sensitivity, modulating several risk factors such as obesity, dyslipidemia, lipid peroxidation, coronary artery abnormalities, and Non-Alcoholic Fatty Liver Disease (NAFD) (14, 15).

**Figure 1:**
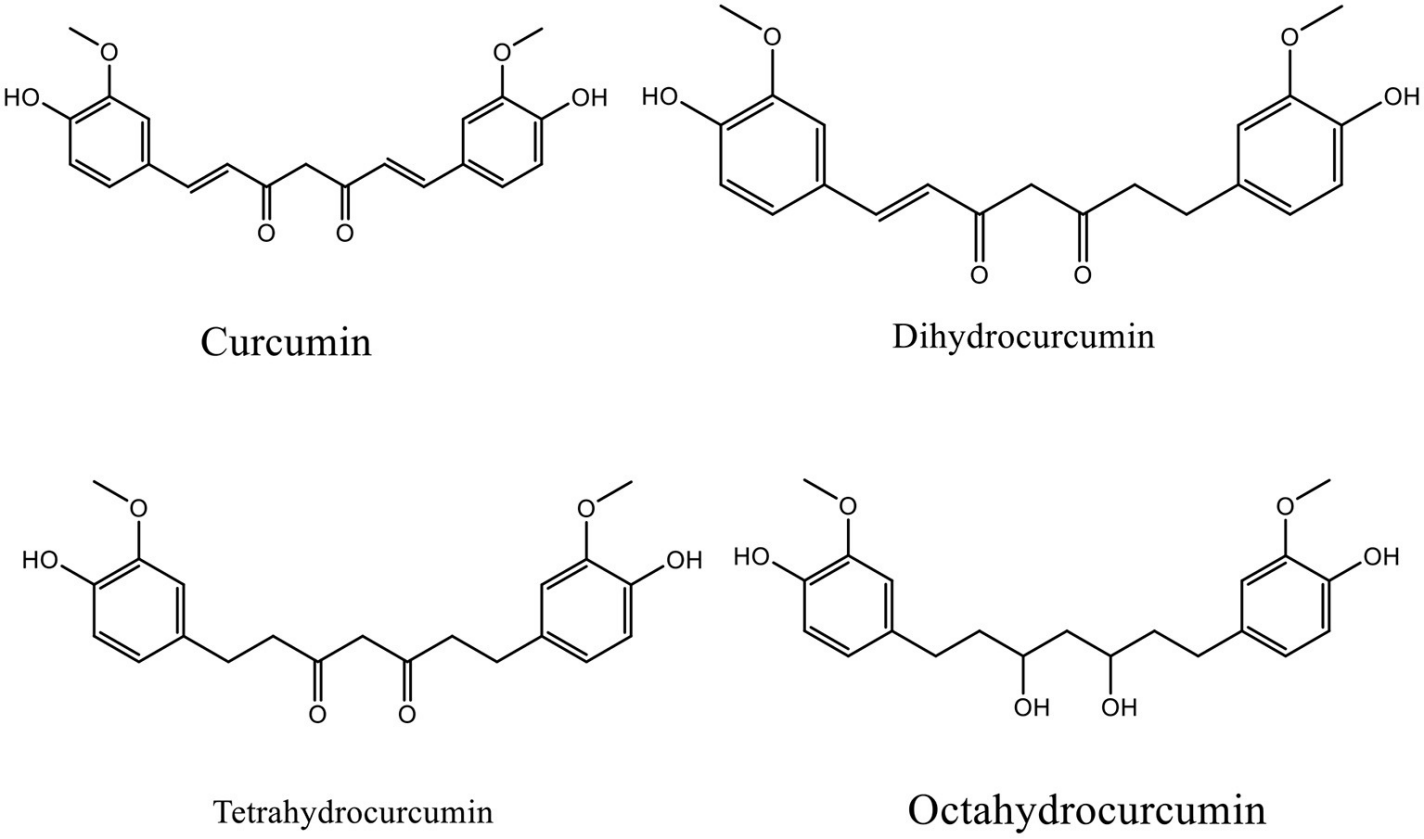
Chemical structure of curcumin and its major metabolites.

Curcumin, a substantial active compound of turmeric (*Curcuma longa*), has multiple pharmacological effects and is safe for food-based biotechnology and medical applications (Figure 1) (16, 17). Curcumin and derivatives can be classified as phenolic secondary metabolite which possess at least two phenolic hydroxyl groups, which can dissociate to negative charged phenolate ions under physiological conditions. Curcumin can thus interact with proteins by forming hydrogen bridges and ionic bonds, which in consequence can alter the properties of a protein (18). Furthermore, as a phenolic phytoconstituent, curcumin has antioxidative properties and can thus counteract the negative consequences of a high concentration of reactive oxygen species (ROS) (19). However, its application is limited by poor bioavailability (20, 21), thus there have been attempts to develop curcumin delivery systems, such as nanoparticles and liposomes, along with some evidence on its efficacy and safety (22–25).

A systematic review published in 2016 reviewed the effects of curcumin on chronic diseases including cancer, inflammatory diseases, metabolic diseases, cardiovascular diseases, neurological disease, and skin diseases (26). More recent clinical studies and systematic review/meta-analysis of curcumin, especially regarding MS, have been published but there is heterogeneity regarding curcumin's effects in MS, mainly based on the phenotypic causes. Tabrizi et al. reported that curcumin reduced fasting glucose, HbA1c, triglycerides, and total cholesterol in MS patients, although no changes in low density lipoprotein (LDL) and high density lipoprotein (HDL) levels were observed (27). In the other hand, Azhadri et al. found that curcumin improved fasting blood glucose, triglycerides, HDL, and diastolic blood pressure without affecting systolic blood pressure or waist circumference (28). Curcumin supplementation can increase circulating adiponectin in patients with MS, reducing the risk of cardiovascular diseases (29) and promote lipid metabolism and glycemic control in polycystic ovary syndrome (PCOS) patients with no significant adverse effects (30).

The rationale for the current review is as follows:

- To identify new possible molecular and cellular targets of curcumin
- To provide a comprehensive updated understanding and meta-analysis of the clinical effects of curcumin on MS and its components
- To understand that the response to curcumin depends on the metabolic profile and phenotypes involved in the disease progression
- To provide information about the effects of “environmental factors” (metabolizing enzymes, gut microbiome, nutrient) on curcumin metabolism and biotransformation that appear strongly correlated with its efficacy and bioavailability and represents a necessary knowledge to move forward personalized interventions and prescriptions

## Molecular Targets of Curcumin

### Fundamental Molecular Targets

**Figure 2:**
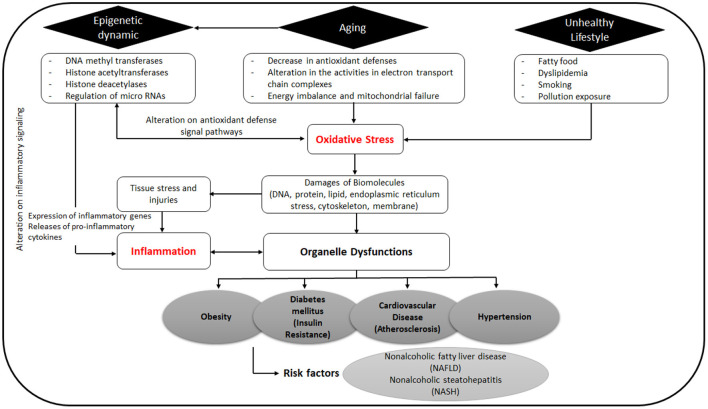
Origin and pathology of oxidative stress and inflammation can lead to metabolic diseases.

**Figure 3:**
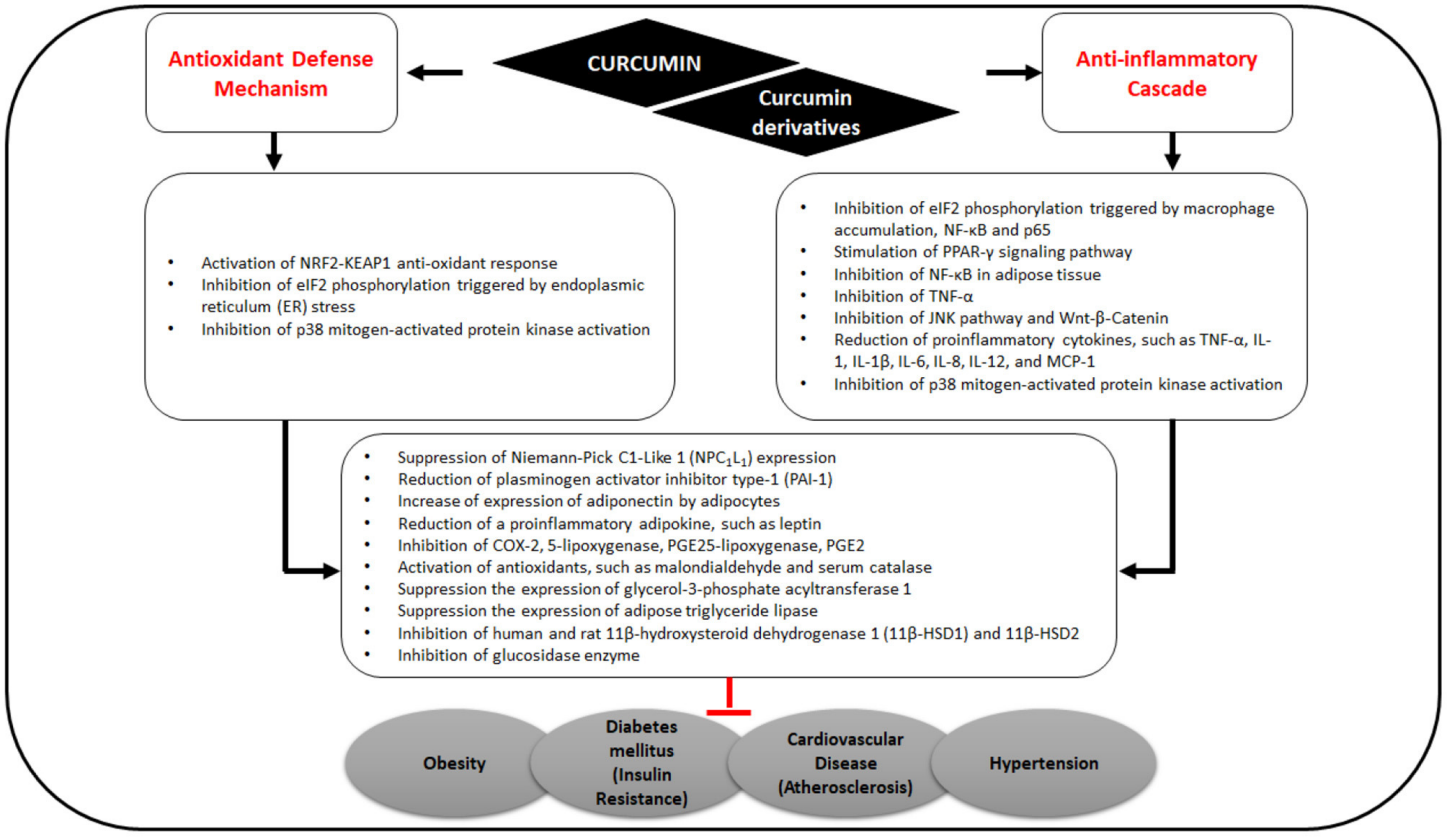
Effects of curcumin on antioxidant defense mechanisms and anti-inflammatory cascade related to MS.

Curcumin interacts with macrophages, muscle cells, adipocytes, hepatic stellate, and pancreatic cells *via* targeting multiple molecular components including transcription factors, inflammatory mediators, enzymes, such as protein kinases (31, 32), histone acetyltransferase (33, 34), DNA methyltransferases, histone deacetylases, and modulation of micro RNAs (Figure 2) (35). Furthermore, many of the protein targets are components of multiple cell signaling pathways involved in the development of metabolic disorders, such as Peroxisome proliferator-activated receptor gamma (PPARγ), nuclear factor κB (NF-κB)/nuclear factor-erythroid 2 related factor 2 (NRF2), Wnt/β-catenin, NRF2/Kelch-like ECH-associated protein 1 (KEAP1), FOXO, and phosphatidylinositol-3-kinase(PI-3-K)/Akt/tumor necrosis factor-α (TNF-α) (Figure 3) (36).

By stimulating PPARγ and NRF2 signaling pathways, curcumin consequently up-regulates adiponectin and other gene products, leading to the downregulation of adipokines including resistin, leptin, TNF, interleukin (IL)-6, and monocyte chemotactic protein-1 (37). In a recent *in silico* study, Shakour et al. reported that curcumin and its derivatives such as bisdemethoxycurcumin, cyclocurcumin, and demethoxy curcumin have an important regulatory function in oxidative and inflammatory processes involved in many diseases (38).

### Curcumin in Inflammation, Obesity, and Diabetes

Obesity is a chronic disease associated with inflammation; it has a main role in MS development as well as increasing the risk of atherosclerosis. Alteration of adipose tissue reduces lipid metabolism, causing an imbalance in the energy storage in fat tissue, thus affecting endocrine system and immune response. These alterations are primarily marked by the secretion of leptin, adipocyte-specific hormone resistin, and plasminogen activator inhibitor type-1 (PAI-1). In addition, inflammatory cytokines are also released such as TNF, IL-1, IL-6, and monocyte chemotactic protein (MCP)-1. These cytokines and chemokines significantly promote insulin resistance and mediate chronic inflammation.

Curcumin in turmeric is notable for its antioxidant and anti-inflammatory effects (Figure 3) (39, 40) and for ameliorating obesity risk factors (41) *via* the inhibition of NF-κB in adipose tissue, thereby reducing the expression of TNF-α, IL-6, MCP-1, PAI-1, and increasing adiponectin expression (40).

Some molecular pathways of inflammation and obesity are cross-talked with insulin resistance and diabetes (42, 43). Oxidative stress induced by elevated levels of circulating free fatty acids, endoplasmic reticulum (ER) stress, and proinflammatory signaling pathways also contribute to the induction of insulin resistance (43–45). Curcumin acts mainly as a signaling modulator that activates endogenous antioxidant responses such as the NRF2-KEAP1 (45) and has been demonstrated to reduce ER stress in a high-fat diet mice model, *in vitro* in primary adipocytes (46), and in palmitate-induced insulin resistance in human umbilical vein endothelial cells (HUVECs) (47). Lipolysis decreases and insulin resistance improves with decreased ER stress (47) but the relationship between ER stress, mitochondrial stress, and inflammation is complex (43). Thus, the curcumin effects require further investigation to elucidate the mechanism and links to other metabolic processes.

### Curcumin in Cardiovascular Diseases

Cardiovascular diseases (CVDs) are the notable cause of death globally. Curcumin has been shown to prevent and treat CVDs *via* modulation of multiple signaling pathways related to cellular proliferation, growth, survival, oxidative stress, and inflammation. Curcumin prevents cardiovascular dysfunction and its primary risk factors (atherosclerosis, aortic aneurysm, myocardial infarction, and stroke) (48, 49) by activating endogenous antioxidant, anti-inflammatory, and anti-apoptotic responses, thereby improving cardiovascular complications, heart failure, and cardiotoxicity (50–52).

## Preclinical Studies

Preclinical studies are a fundamental stage in drug development in which essential pharmacologic features of drug candidates are revealed before translating into clinical studies (53, 54). Investigations of pharmacodynamics and pharmacokinetics determine how the body responds to the drug also elucidate potential toxicity (55). It is vital to consider the disease origin and pathology to select an appropriate model, either cells or animals; however developing animal models to mimic MS is challenging (56, 57) as this multifactorial disease involves various enzymes and biomolecules in several organs. Also, interconnected organs can potentially result in a complication, thus optimizing appropriate models to understand the effect of curcumin on specific sites, therapeutic dosage, and administration route is critical. Table 1 presents a summary of the preclinical models used to study the effect of curcumin on MS, with dose administration noted to be a critical aspect resulting in different therapeutic effects.

**Table 1:** Summary of *in vitro* or animal models studies on the effect of curcumin and its analogs on the individual parameter of MS.

**Compound**	**Model**	**Dose**	**Mechanism of action**	**References**
Curcumin	3T3-L1 preadipocytes	High dose: >30 μM	High dose curcumin generates preadipocyte apoptosis in a time- and dose-dependent manner and caspase-dependent pathways (3-, 8-, and 9-)	(58)
		Low dose: <15 μM	Low dose curcumin suppresses adipocyte differentiation *via* modulation of cell cycle regulator expression, downregulating PPAR, and CCAAT/enhancer-binding protein (C/EBP) expression, blocking differentiation medium-induced catenin downregulation and lowering lipid accumulation	
Curcumin	Male Wistar rats with diet-induced MS	High dose: curcumin suspension 100 mg/Kg/day	High dose curcumin nanoparticles may reduce cardiac injury and improve inflammation of ventricular fibrosis	(59)
		Low dose: curcumin nanoparticles 5 mg/Kg/day	Low dose curcumin nanoparticles lower blood pressure, target Uncoupling Protein (UCP) 2 and induce vascular tone and the predisposition to vascular disease; reduce inflammation in white adipose tissue and increase energy expenditure *via* activation of brown adipose tissue	
Curcumin	Obese C57BL/6 J mice	After 16 weeks of a Western-style diet, curcumin accumulates in eWAT (299 ± 113 pmol/g)	Curcumin accumulates in eWAT and inhibits eukaryotic translation initiation factor 2 (eIF2) phosphorylation, which is triggered by ER stress, macrophage accumulation, NF-KB p65, and leptin but not TNF- and IFN- levels. Curcumin reduces lipogenesis and lipolysis by suppressing the expression of glycerol-3-phosphate acyltransferase 1 and adipose triglyceride lipase, resulting in a decrease in diacylglycerols (DAGs) and DAG-derived glycerophospholipids.	(60)
Curcumin	Skeletal muscle C2C12 cells	5, 20, and 40 μM	Curcumin exhibits anti-inflammatory activity in C2C12 cells *via* suppressing the JNK and NF-KB pathways and reducing oxidative stress	(61)
Curcumin	PCOS -induced Wistar rats	100 and 300 mg/Kg	Curcumin modifies the lipid profile and increases insulin sensitivity	(62)
Curcumin	High fructose diet-induced adult male Sprague Dawley rats	200 mg/Kg/day	Curcumin exhibits antioxidant, antiinflammatory, antihyperglycemic, anti-hypercholesterolemic, anti-hypertriglyceridemic, and antihyperuricemic, weight loss, and blood pressure-lowering effects in high fructose diet-induced rats *via* the activation of antioxidants, such as malondialdehyde and serum catalase, and suppression of inflammatory factors, such as TNF-α and NF-KB	(63)
Curcumin	Fructose diet and STZ-induced diabetes in adult male Wistar rats	1 g/Kg	Curcumin pre-treatment improves metabolic changes (hyperglycemia, hypercholesterolemia, hypertriglyceridemia) and oxidative stress in rats induced by both fructose-induced MS and STZ-induced diabetes, as well as lowering systolic blood pressure due to its ability to reverse oxidative stress	(64)
Curcumin	Male Sprague Dawley rats	200 mg/Kg/day	Curcumin competitively inhibits human and rat 11β-hydroxysteroid dehydrogenase 1 (11β-HSD1), with selectivity against 11β-HSD2. In high-fat-diet-induced obese mice, curcumin lowered serum glucose, cholesterol, triglycerides, and low density lipoprotein levels. Inhibition of 11β-HSD1 is substantially more potent with four curcumin derivatives and (1E,4E)-1,5-bis(thiophen-2-yl) penta-1,4-dien-3-one (compound 6), has a much lower IC_50_ compared to the parent compound.	(65)
Curcumin with piperine and quercetin	Albino female Wistar rats	100 mg/Kg/day	Curcumin combined with piperine and quercetin enhances peripheral glucose utilization to decrease blood glucose levels, possibly *via* inhibition of glucosidase enzyme, and can also increase the pancreatic insulin output	(66)
Curcumin	Caco-2 cells	40–50 μM	Curcumin reduces cholesterol absorption *via* suppression of Niemann-Pick C1-Like 1 (NPC_1_L_1_) expression	(67)
Curcumin analog (Curcumin5-8)	High-fat diet-induced obesity C57BL/6 mice	100 mg/Kg/day	Compared to the parent curcumin, curcumin5-8 inhibits fatty acid synthesis and lipid droplet formation to inhibit fatty liver formation. The improved NAFLD and hepatic triglyceride levels are not associated with increased autophagy, as oxidative stress is suppressed consequently reducing the risk of metabolic diseases, such as obesity, fatty liver, and diabetes. Compared to the untreated group, curcumin 5–8 significantly improves insulin resistance and shows a hepatoprotective effect against lipid toxicity and apoptosis, with decreased serum alanine aminotransferase levels	(68)

## Meta-Analysis of Curcumin in Metabolic Syndrome

Due to the divergence of clinical evidence of curcumin in improving MS, we included up-to-date meta-analyses in the current review to precisely estimate the effect size. Heterogeneity value in meta-analyses is our main concern to analyze and synthesize rigorous conclusions. We specify the heterogeneity data based on several MS parameters to provide parameter-specific effectiveness of curcumin. Eighteen meta-analyses from Randomized Controlled Trials (RCT) were evaluated (Table 2) showing high heterogeneity on some parameters (I2 > 50%, *P* < 0.05), indicating that curcumin may partially reduce MS through a specific mechanism.

**Table 2:** Meta-analysis of RCT of curcumin, curcuminoids, or turmeric in patients with triggering components in the origin of the MS (27, 28, 69–83).

**Author**	**Diseases/** **Focuses**	**Design Study**	**Parameter Outcome**	**Significance on measured parameter**	**Heterogeneity result from meta-analysis**	**Subgroup analysis results**
Sahebkar (80)	Healthy subjects and patients with chronic inflammatory disease	Systematic review and meta-analysis involving 8 RCTs	Total cholesterol LDL-C Triglycerides HDL-C	Not significant Not significant Not significant Not significant	I^2^ = 98%; *P* < 0.00 I^2^ = 99%; *P* < 0.00 I^2^ = 0%; P = 0.96 I^2^ = 90%; *P* < 0.00	Subgroup analysis of subjects with cardiovascular risk indicating similar significance on lipid profile, all with high heterogeneity, except for HDL-C (I^2^ = 0%)
Qin et al. (81)	Chronic inflammatory disease, including metabolic syndrome	Systematic review and meta-analysis involving 7 RCTs	LDL-C ↓^*^ Triglycerides ↓ Total Cholesterol ↓ HDL-C	Significant Significant Significant Not significant	I^2^ = 42,1%; *P* < 0.125 I^2^ = 24.5%; *P* < 0.242 I^2^ = 73.8%; *P* < 0.054 I^2^ = 0,0%; *P* < 0.705	• Triglycerides did not change significantly in the turmeric powder subgroup compared to the mixture subgroup • In the hyperglycemia subgroup, there was no significant triglycerides reduction compared to the MS subgroup • All subgroup analyses reported high heterogeneity
de Melo ISV et al. (77)	Dysglycemia	Systematic review and meta-analysis, with 11 RCTs included	Fasting plasma glucose ↓ HbA1c ↓^*^	Significant Significant	All of the findings observed with high heterogeneity (I^2^ >50%, *P* < 0.05)	• Fasting plasma glucose did not change significantly in non-diabetic subjects compared to subjects with prediabetes, diabetes, or MS • Fasting plasma glucose did not change significantly when using turmeric supplementation compared to curcumin and curcuminoids • HbA1c did not change significantly when using curcuminoids supplementation compared to curcumin supplementation • All subgroup analyses had high heterogeneity, except for fasting plasma glucose in non-diabetic subjects • Baseline blood glucose affected treatment effectiveness, the higher blood glucose baseline, the more effective the curcumin treatment
Mousavi et al. (82)	Chronic inflammatory disease including MS	Meta-analysis, involving 11 RCTs	Body weight ↓	Significant BMI ↓^*^ Waist circumference	All of the findings observed with high heterogeneity (I^2^ >50%, *P* < 0.05) Significant Not significant	• Curcumin dose of 1,000 mg/day for ≥8 weeks duration significantly reduced body weight and BMI in overweight subjects • All subgroup analyses had high heterogeneity
Qin et al. (70)	Chronic inflammatory disease including MS	Meta-analysis, involving 8 RCTs	MDA ↓^*^ SOD ↑^*^	Significant Significant	All of the findings observed with high heterogeneity (I^2^ >50%, *P* < 0.05)	• The combination of curcuminoids and piperine lowered MDA • There is no significant difference in the improvement of MDA and SOD regardless of the associated disease, dose, and duration of administration • All subgroup analyses had low heterogeneity, except for osteoarthritis with regards to MDA level
Tabrizi et al. (27)	MS and its components, NAFLD, and coronary vascular artery	Systematic review and meta-analysis, 15 RCTs were included	IL-6 ↓^*^ hscrp ↓^*^ MDA ↓^*^ TNF-alpha SOD Fasting plasma glucose ↓	Significant Significant Significant Not Significant Note Significant Significant	All of the findings observed with high heterogeneity (I^2^ >50%, *P* < 0.05)	• None
Tabrizi et al. (27)	MS and its components, NAFLD, and coronary vascular artery	Systematic review and meta-analysis involving 26 RCTs	Fasting plasma glucose ↓ HbA1c ↓^*^ HOMA-IR ↓^*^ Triglycerides ↓ Total cholesterol ↓ Insulin ↑	Significant Significant Significant Significant Significant Significant Not Significant	All of the findings observed with high heterogeneity (I^2^ >50%, *P* < 0.05)	• Curcumin in a dose of >500 mg/day supplemental dose for ≤ 8 weeks significantly improved total cholesterol and LDL-C in patients with a BMI ≥27, intervention subgroup • All subgroup analyses had high heterogeneity
			LDL-C HDL-C	Not Significant		
Akbari et al. (84)	MS and its components	Systematic review and meta-analysis involving 18 RCTs	BMI ↓^*^ Waist circumference ↓ Weight ↓ Leptin ↓ Adiponectin ↑ Hip ratio	Significant Significant Significants Significant Significant Not Significant	I^2^ = 69,7%; P = 0.000 I^2^ = 45,7%; P = 0.027 I^2^ = 42,1%; P = 0,087 I^2^ = 0,0%; P = 0,613 I^2^ = 94,5%; P = 0,000 I^2^ = 0,00%; P = 0,792	• BMI, body weight, waist circumference, leptin, and adiponectin are consistently significant with >500 mg/day dose but some results (toward BMI and adiponectin) had high heterogeneity • BMI, body weight, waist circumference, leptin, and adiponectin showed various results regarding intervention duration, all with high heterogeneity, except for leptin • The hip ratio is not significantly affected by curcumin in all subgroups (regarding dose and duration) with low heterogeneity • Leptin is significantly affected in all subgroups (regarding dose and duration) with low heterogeneity
Azhdari et al. (28)	MS	Systematic review and meta-analysis involving 7 RCTs	Fasting plasma glucose ↓ Triglycerides ↓ HDL-C ↑^*^ SBP DBP ↓^*^ Waist circumference	Significant Significant Significant Not Significant Significant Not Significant	I^2^ = 90.1%; P = 0.00 I^2^ = 94.4%; P = 0.00 I^2^ = 98.6%; P = 0.00 I^2^ = 48.2%; P = 0.145 I^2^ = 48.7%; P = 0.142 I^2^ = 0.00%; P = 0.595	• No available subgroup analysis due to lack of trials
Clark et al. (26)	Prediabetes and type 2 diabetes mellitus	Systematic review and meta-analysis involving 6 RCTs	Adiponectin ↑	Significant	I^2^ = 87.2%; P = 0.00	• Curcumin significantly improved adiponectin in <10-week intervention duration subgroup (I^2^ = 49,5%)
Hadi et al. (78)	Improvement of blood pressure parameters	Systematic review and meta-analysis, 11 RCTs were included	SBP DBP	Not significant, with only−1.24 mmHg reduction Not significant	I^2^ = 0% I^2^ = 1%	• Significant SBP lowering effect with ≥12-week intervention, without clinically significant effect (overall only 1,24 mmHg reduction of SBP)
Huang et al. (76)	Chronic inflammatory disease, including MS	Meta-analysis, involving 14 RCTs	Fasting plasma glucose ↓ HbA1c ↓^*^ HOMA-IR ↓^*^	Significant Significant Significant	All findings had high heterogeneity (I^2^ >50%, *P* < 0.05)	• Significant results of improved fasting plasma glucose and HbA1c were found in patients with diabetes treated with ≥300 mg/day for ≥12 weeks • All subgroup analyses had low heterogeneity, except for HbA1c correlation in the ≥12 weeks subgroup
Simental-Mendía LE et al. (79)	Healthy subjects and patients with chronic inflammatory disease	Systematic review and meta-analysis involving 20 RCTs	Triglycerides ↓ HDL-C ↑^*^ Total cholesterol LDL-C	Significant Significant Not Significant Not Significant	I^2^ = 65.55% I^2^ = 37.24% I^2^ = 84.25% I^2^ = 85.64%	• No significant difference was observed regarding the duration of curcuminoid supplementation on all lipid parameters • There was no significant effect of curcumin on HDL-C in both of <12-week and ≥12-week intervention subgroups
White et al. (72)	Chronic inflammatory disease, including MS, and cardiovascular diseases	Systematic review and meta-analysis, involving 19 RCTs	CRP, hscrp, IL-1β, IL-6, and TNF-α^*^	Not significant	All findings had high heterogeneity (I^2^ >50%, *P* < 0.05), except for hsCRP (I^2^ = 21%)()	• No conclusive heterogeneity source, the details were not mentioned
Yuan et al. (83)	Chronic inflammatory disease, including	Systematic review and meta-analysis involving 16 RCTs	Triglycerides ↓ Total Cholesterol ↓ LDL-C ↓^*^ HDL-C ↑^*^	Significant Significant Significant Significant	I^2^ = 75%; *P* < 0.00 I^2^ = 48%; *P* < 0.00 I^2^ = 71%; *P* < 0.00 I^2^ = 70%; *P* < 0.00	• More significant reduction in triglycerides observed in ≥1,000 mg/day dose than the low dose (I^2^ = 77% in high dose vs. 43% in low dose group)
	metabolic syndrome					• Higher reduction of LDL-C was found in ≥300 mg/day supplemental dose compared to low dose (I^2^ = 55% vs. 80%) • The effectiveness of triglyceride reduction was observed at 24-week, 12-week, and 8-week intervention, therefore, triglyceride might be lowered with a minimum of 8-week intervention (I^2^ <50%) • There was no significant improvement in triglyceride, LDL-C, HDL-C, and total cholesterol at 4-weeks. LDL-C and total cholesterol-lowering effect required 12-week intervention (I^2^ = 0 for all measurements) • There was no significant improvement of HDL-C with 8-week intervention duration, low dose (≥300 mg/day), and in non-diabetic subjects (I^2^ = 53% vs. 0% vs. 53% respectively) • There was heterogeneity among the subgroup analyses. All blood lipid results had low heterogeneity with a minimum 12-week intervention duration. High dose intervention and subjects with diabetes type 2 had lower total cholesterol with low heterogeneity.
Altobelli et al. (75)	Uncomplicated type 2 diabetes	Meta-analysis involving 7 RCTs	HbA1c ↓^*^ HOMA-IR ↓^*^ LDL-C ↓^*^ BMI HDL-C Triglycerides ↓ Total Cholesterol ↓	Significant Significant Significant Not Significant Not Significant Significant Significant	I^2^ = 42.42%; P = 0.107 I^2^ = 0.00%; P = 0.916 I^2^ = 0.00%; P = 0.083 I^2^ = 0.00%; P = 0.514 I^2^ = 0.00%; P = 0.116 I^2^ = 41.56%; P = 0.144 I^2^ = 0.00%; P = 0.573	• Low heterogeneity, no need to subgroup
Ferguson et al. (69)	Healthy subjects and patients with chronic inflammatory disease	Systematic review and meta-analysis involving 32 RCTs	CRP ↓^*^	Significant	I^2^ >50%, *P* < 0.05	• Bio-enhanced curcuminoids led to the greatest reduction of CRP, followed by non-bio-enhanced curcuminoids, bio-enhanced curcumin, curcumin (without regards to bio-enhancement), and turmeric consecutively. Regarding CRP measurement, the only low heterogeneity result was observed for the curcuminoid non-bio-enhanced group.
			IL-6	Significant	I^2^ >50%, *P* < 0.05	• Bio-enhanced curcuminoids led to the greatest reduction of IL-6, followed by non-bio-enhanced curcuminoids, bio-enhanced curcumin, and curcumin consecutively. Turmeric had no effect and curcumin (regardless of bio-enhancement) and bio-enhanced curcumin significantly lowered IL-6 with low heterogeneity, meanwhile, other results had high heterogeneity.
			TNF-α^*^	Significant	I^2^ >50%, *P* < 0.05	• Bio-enhanced curcuminoids led to the greatest reduction of TNF-α compared to bio-enhanced curcumin
Gorabi et al. (73)	Inflammatory diseases, including MS and its components	Meta-analysis involving 32 RCTs	IL-1 ↓^*^ TNF-α ↓^*^ IL-6 IL-8	Significant Significant Not Significant Not Significant	All findings had high heterogeneity (I^2^ >50%, *P* < 0.05)	• TNF-α was significantly decreased with a curcumin dose of <1,000 mg/day • TNF-α was significantly decreased with ≥10 weeks of curcumin supplementation • All subgroup analyses had high heterogeneity

*CRP, C-Reactive Protein; hs-CRP, high-sensitivity C-reactive protein; IL-1, Interleukin 1; TNF-α, Tumor Necrosis Factor – α; IL-6, Interleukin 6; IL-8, Interleukin 8; MDA, malondialdehyde; SBP, Systolic Blood Pressure; DBP, Diastolic Blood Pressure; HbA1c, glycosylated hemoglobin; SOD, superoxide dismutase; LDL-C, Low Density Lipoprotein 264 Cholesterol; HDL-C, High Density Lipoprotein Cholesterol; BMI, Body Mass Index; HOMA-IR, Homeostatic Model Assessment of Insulin Resistance*.

### Curcumin Effects on Inflammation

Curcumin is a popular traditional medicine for inflammatory conditions (39). Inflammation is involved in MS as elevated visceral adipose tissue modulates adipokine secretion leading to decreasing adiponectin levels, increasing leptin and proinflammatory cytokines (i.e. TNF-α and IL-6), thereby promoting endothelial cell dysfunction and atherosclerosis (85). Non-bio-enhanced curcuminoid can lower C-Reactive Protein (CRP), while other forms of curcumin, such as bio-enhanced curcuminoids, bio-enhanced curcumin, curcumin without bio-enhancement, and turmeric had no effect, showing the tendency of curcumin to lower IL-6 regardless of bio-enhancement (69). Qin et al. (70) also showed that malondialdehyde (MDA), a stress oxidative marker, is lowered by curcumin regardless of intervention dose, duration, and underlying disease, except for osteoarthritis. Moreover, the combination of curcuminoids and piperine is more effective than curcumin alone to reduce MDA levels (70) but does not affect TNF-α, leptin, or adiponectin (27, 69, 71–74, 84). Consumption of nanoparticles of curcumin for 6 weeks, along with regular exercise in 44 older women with MS, decreased IL-6, increased IL-10, and increased brain-derived neurotrophic (BDNF) level (86).

### Curcumin Effects on Obesity

Curcumin has been reported to activate AMP-activated protein kinase (AMPK) to inhibit adipocyte differentiation. Stimulation of AMPK down-regulated PPAR-γ in 3T3-L1 adipocytes to prevent insulin resistance and obesity (87). Di Piero et al. reported that curcumin has a beneficial effect on weight management in 44 overweight subjects by improving weight loss, reducing body fat, increasing waistline reduction, improving hip circumference reduction, and enhancing reduction of BMI (p < 0.01 for all comparisons) without significant statistical effect on phosphatidylserine (88). Akbari et al. (84) reported that curcumin in a dose of 70–2,400 mg/day for 4–36 weeks can significantly reduce BMI and body weight compared to placebo. The effect of curcumin based on dose and duration of intervention, however, showed high heterogeneity among clinical studies. Waist circumference was also significantly reduced but there was no clinical data regarding a clinical significance in the hip ratio (84). Azhdari et al. and Altobelli et al. also reported the insignificant effect of curcumin in reducing BMI and waist circumference (28, 75), thus the effectiveness of curcumin in improving obesity is unclear.

### Curcumin Effects on Diabetes Mellitus and Insulin Resistance

Diabetes mellitus (DM) is a metabolic disorder with hyperglycemia as the main clinical feature. The association between insulin and its producing organ, the pancreas, forms the fundamental understanding of the diseases. Over the last 100 years, clear evidence has distinct two types of diabetes: “insulin-dependent” (Type 1–T1DM) in which the pancreas completely fails to produce insulin and “non-insulin-dependent” (Type 2–T2DM). In T2DM, insulin hypersecretion by β-cell (hyperinsulinemia) causes the inability of the pancreas to cope with required insulin and later can experience insulin resistance, resulting in elevated blood glucose levels (hyperglycaemic hyperinsulinemia). Insulin resistance, as an onset of hyperinsulinemia, can cause the inability of β-cell to secrete insulin, apparently due to the manifestation of hyperglycemia (hyperglycaemic hypoinsulinemia). In addition, the liver contributes to systemic insulin resistance in which gluconeogenesis is undertaken, and hepatic insulin resistance can occur during the hyperglycemia state. Muscle insulin resistance can also occur when fat accumulation disturbs glucose uptake and affects insulin signaling. Fat accumulation in pancreatic cells causes β-cell dysfunction, increases plasma glucose, and reduces insulin response. Understanding the complex pathologies of DM promotes the evidence that DM is associated with greater risk factors related to cardiovascular diseases, such as coronary heart diseases and stroke, and non-vascular diseases, including cancer, neuro disorder, and liver diseases (89).

Nine-month curcumin consumption improves β-cell function by increasing Homeostatic Model Assessment (HOMA)-β, lowering Homeostatic Model Assessment of Insulin Resistance (HOMA-IR) and C-peptide (90). In the meta-analysis by Huang et al. (76), curcumin significantly improved fasting plasma glucose, HOMA-IR, and glycated hemoglobin (HbA1c). Subgroup analysis confirmed the significant effects of curcumin (≥300 mg/day dose and ≥12-week intervention) in improving plasma fasting glucose and HbA1c in the diabetes subgroup, with no significant effects in the non-diabetic subgroup (76). Melo et al. also reported that curcumin treatment was more effective in the higher blood glucose baseline (77). Hodaei et al. confirmed the findings reporting daily administration of 1,500 mg curcumin has a beneficial influence in decreasing weight and fasting blood glucose in subjects with type 2 diabetes (91).

### Curcumin Effects on Hypertension

Hypertension is a common component of MS and there are two meta-analyses of curcumin in hypertension. Hadi et al. reported no significant effect of curcumin on systolic blood pressure (SBP) and diastolic blood pressure (DBP), although SBP was reduced (average reduction of 1.24 mmHg) but not significantly at ≥ 12-week (78). Azhdari et al. confirmed that curcumin has no significant effect on SBP but significantly improved DBP (heterogeneity <50%) (28).

### Curcumin Effects on Dyslipidemia

Dyslipidemia is a clinical condition described as elevated total or LDL-cholesterol (LDL-C) or low HDL cholesterol (HDL-C) levels and is considered a serious risk factor for coronary heart disease and stroke (92). Yuan et al. (83) reported that curcumin might reduce total cholesterol when administrated to diabetic patients for a minimum of 12 weeks (>300 mg/day), reduce LDL-C after a minimum 12-week intervention, and triglycerides after a minimum 8-week intervention. More prolonged administration may be required to improve the efficacy of curcumin on dyslipidemia. However, Simental-Mendía et al. found that curcumin did not affect HDL-C regardless of intervention duration (<12-week and ≥12-week) (79). Another meta-analysis also confirmed the non-significant effect of curcumin in improving HDL-C with low heterogeneity (75, 80, 81).

## Other Clinical Studies

Apart from the meta-analysis included in this review, other studies were conducted on subjects not specifically on the MS.

### Curcumin Effects on Non-alcoholic Fatty Liver Disease (NAFLD)

NAFLD and MS are two different disorders with overlapping clinical and pathophysiological characteristics. NALFD indicates mere liver steatosis to overt liver cirrhosis (93). Oxidative stress and inflammation play major roles in the progression of NAFLD. As an anti-inflammatory modulator, curcumin might improve health parameters related to NAFLD (94). Some RCT reported the effectiveness of curcumin and curcuminoids in improving various health parameters in NAFLD patients, i.e., serum levels of pro-inflammatory cytokines (IL-1α, IL-1β, IL-2, IL-4, IL-6, IL-8, IL-10, TNF-α, monocyte chemoattractant protein-1, interferon γ, vascular endothelial growth factor, and epidermal growth factor, fasting plasma insulin (FPI), HOMA-IR, liver transaminases, fatty liver index, serum cortisol, ultrasound morphological of the liver, and other parameters included in MS (*p* < 0.05) (94–112). The findings were consistent regarding the beneficial effects on improving NAFLD risk factors (113), with only one study having conflicting results, whereby curcumin was reported to have no impact on liver transaminases, metabolic and inflammatory parameters. This inconsistency is probably due to the low curcumin dose of only 35 mg curcumin per intake, thus reducing its bioavailability and effectiveness (114). The combination of curcumin with other substances, such as ursodeoxycholic and piperine, has shown a positive outcome in improving NAFLD (115–117). Dose and term administration should be critically appraised.

### Curcumin Effect on Sleep Duration

Sleep duration might be linked to MS and a short sleep duration has been associated with obesity. Sleep deprivation could increase oxidative stress mediators in the brain and since curcumin may relieve oxidative stress, it may be beneficial in suppressing stress and prolonging sleep duration. However, according to a trial by Saberi-Karimian et al. curcumin did not have any impact on sleep duration in patients with MS (118).

### Curcumin Effect on Oxidative Stress and Heat Shock Proteins

Serum copper and zinc and the ratio of Zn/Cu are often used to identify oxidative stress and inflammation (119, 120). Furthermore, protein chaperones, such as heat shock proteins (HSP) are highly expressed during oxidative stress (121). Some recent studies reported the effects of curcumin and its derivatives on some oxidative stress markers in MS patients (122), for instance, serum copper, zinc, and trace elements are elevated in patients with MS compared to healthy subjects (120). A double-blind clinical trial of 120 subjects with MS reported increased serum zinc and Zn/Cu ratio after administration of curcumin and phospholipid curcumin complex at a dose of 1 g/day for 6 weeks (123). However, Mohammadi et al. reported no significant difference in the alteration of HSP 27 serum concentrations among the study groups (group received curcumin, phospholipid -curcumin complex, or a placebo for 6 weeks) (124). Oxidative stress occurs as a result of an imbalance between pro-oxidant and antioxidant defense systems, resulting in cellular damage and associated diseases including MS. The redox stress index indicates the pro-oxidant/antioxidant balance in human serum due to oxidation and reduction reactions that coincide (125). Ghazimoradi et al. (126) conducted a double-blind, randomized, placebo-controlled trial over 6 weeks of 120 individuals with MS and three groups of subjects received curcumin, phospholipid curcumin, and placebo. Serum PAB was elevated significantly in the curcumin group. Still, the study did not assess different doses of curcumin and did not consider the impact of other adjuvants and its possible interaction that may affect the efficacy of curcumin (126). No significant effect was observed on the serum antioxidant vitamin E level after administration of 1 g curcumin supplement per day for 6 weeks, indicating that the reported antioxidant effect of curcumin is mainly *via* the modulatory action on the intracellular antioxidant system rather than serum vitamin E (127).

### Clinical Studies of Nano Curcumin

One of the limitations of using phytochemicals often is their poor bioavailability in the human body, thus nano formulations have been employed to improve the delivery of phytochemicals such as curcumin, berberine, naringenin, quercetin, emodin, myricitrin, stevioside, and resveratrol to the target organ and cells (128). Nano-based formulations for the MS that are biodegradable, biocompatible, nontoxic, and stable after oral administration should be developed focusing on the multifactorial effects rather than a single factor (128). A recent study by Bateni et al. reported that nano-micelle curcumin supplement at a dose of 80 mg/day significantly improved serum triglycerides in patients with MS, although no significant difference was observed in anthropometric measurements, blood pressure, and several biochemical factors (129). Nano curcumin supplementation (80 mg/day for 6 weeks) along with regular exercise significantly reduced inflammation in an elderly woman with MS, as well as decreased the percentage of fat and IL-6, and increased IL-10 and BDNF (86).

### Biosafety of Curcumin

Curcumin had been approved as a safe agent under the Generally Recognized as Safe (GRAS) category by the FDA (130). Nevertheless, the European Medicine Agency (EMA) released a revised version of the guideline for using *C. longa* with special warnings and precautions for patients with obstruction of the bile duct, cholangitis, liver disease, gallstones, and any other forms other biliary diseases. Due to a lack of data on children, adolescents under 18 years, pregnancy, and lactation, the use of *C. longa* is not recommended to those groups of the population (131). Mild undesirable side effects such as dry mouth, flatulence, and gastric irritation should also be critically appraised (131). Panahi et al. reported curcumin is well tolerated with the possibility of minor side effects, including diarrhea, constipation, headache, skin rash (132). A clinical study also reported nausea and colds in the curcumin group (20).

Acceptable Daily Intake (ADI) for curcumin as food additive defined by the European Food Safety Agency (EFSA) is 3 mg/kg BW/day based on No Observed Adverse Effect Level (NOAEL) of 250–320 mg/kg BW/day from the toxicity study (133). Some studies reported curcumin is considered safe even at high doses of up to 4–8 g/day (40, 134). Rigorous studies to understand curcumin's safety as a single modality and/or combination with other substances are warranted, including human trials, to repulse possible hepatotoxicity and genotoxicity disputes (135–137).

### Ongoing Trials

There are currently six ongoing trials examining the efficacy of curcumin in improving MS parameters, including five trials assessing blood glucose, four trials evaluating serum lipid profile, three trials measuring blood pressure, two trials examining body weight, and three trials evaluating oxidative stress and inflammation markers. Due to the fast metabolism and low gut absorption of curcumin, three of these trials are also examining specific curcumin formulations for enhanced bioavailability, e.g., micellar curcumin, nano curcumin, and nano micelles. Another trial is also investigating the effects of curcumin in combination with other agents including inulin, resveratrol, and omega-3 fatty acids to enhance its effectiveness. One study in Thailand is recruiting many participants (*n* = 200) with a long intervention duration (12 months) of regular curcumin that anticipates that the long-term supplementation with this new curcumin formulation may result in a better outcome (138).

## Metabolomics and Personalized Treatment

Personalized medicine or treatment offers a potential prospect for better patient care by tailoring therapy based on the genotype and phenotype profiles of the patient and diseases progression with greater precision (139, 140). The approach includes earlier diagnoses, risk assessments, and choosing optimum treatment (139). Analysis of metabolic phenotype was introduced more than a decade ago to distinguish health and diseases (141, 142) but its application in intensive care only recently began following the emergence of personalized treatment (143). The application of metabolomics is particularly promising for MS, a syndrome characterized by complex human body metabolites, environmental and genetic factors. Some exploratory experiments recently reported the metabolic profile of MS in which some biomarkers have been elucidated for pathogenesis diagnostic, disease progression, and better management (targeted therapy) (144–147).

Since MS is multifaceted, identifying metabolomics and lipidomics is crucial as a comprehensive strategy to manage the significant heterogeneity of metabolites in biofluids. However, this will require appropriate sample preparation, the complexity of data analysis, and new biomarker discovery. The identification of MS biomarkers in adults and the elderly are also worthy of investigation as currently many limitations exist (146). Using this approach, sub-typing pathophysiology of MS offers stratified management, including the prevention and co-treatment using a natural product such as curcumin.

Besides elucidation of phenotype and sub-phenotype of diseases, pharmacometabolomics can provide information regarding how an individual will respond to a drug. Worth noting that the genome, the gut microbiome, sex, nutrition, age, stress, health status, and environment influence the individual response to pharmaceutical compounds, including herbal and phytomedicines. Real-time metabolomic profiling during the treatment could discern the drug mechanism of action, variation in the pharmacokinetic response and determine better-targeted therapy. Pharmacometabolomics in phytomedical clinical research has been considered to aid clinical translation and inform utilization of phytomedicines, most importantly, to be a personalized treatment (148, 149).

Pharmacometabolomics apprise the advance of pharmacogenomics (150), so metabolic profiling can be employed synergistically with genomic analyses since systems biology integrates both genetic and environmental factors. Conversely, employing genetic information only (pharmacogenomics) may not develop into a comprehensive disease phenotyping and therapeutic response, hence both approaches should be implemented simultaneously.

Metabolomics and pharmacometabolomics are particularly necessary to advance the utilization of natural products, such as curcumin (106), as natural products can undergo biotransformation during absorption and metabolism in the body, for instance, the presence of the metabolizing enzyme can alter the activity of the natural products. The intestine microbiome also plays a vital role in the body's response to any drug or nutrition.

### Biotransformation of Curcumin

Curcumin is unstable with low bioavailability (20, 21). There is much evidence that the pharmacological effects of curcumin are mainly due to its metabolites (151). Holder et al. suggest that curcumin elimination will mainly undergoes through bile: The primary biliary metabolites of curcumin consist of the two glucuronide conjugates [tetrahydrocurcumin (THC) and hexahydrocurcumin (HHC)], whereas the minor metabolites consist of ferulic acid and dihydro ferulic acid (152). Glucuronide and sulfate derivatives are the main detectable metabolites in urine and after five days, about 40% are excreted in an unchanged form. Another *in vivo* study indicated that less than half of the curcumin remains in the lower part of the gut, attributed to the biotransformation of curcumin to other components in the gut (153). Kunnumakkara et al. reported that curcumin is undetectable in the heart, blood, and at a very low concentration in the liver, kidney, and portal blood (154).

The intravenous administration of curcumin in rats was associated with a low concentration of THC and dihydrocurcumin (DHC) in plasma. Curcumin was detected in the kidney, liver, and brain. In contrast, THC and DHC were detected in only the kidney and liver, indicating that curcumin crosses the blood-brain barrier, converting a prodrug molecule and possibly targeting neuron dysfunction and disorder (155).

Some investigations in human and rat intestinal tissues showed that curcumin is metabolized in the human intestinal tissue. The extent of curcumin conjugation in the human intestine is much higher than in the rat intestine, however, there is less curcumin conjugation in the human liver compared to rat liver, indicating that curcumin is biotransformed into THC and DHC, thereafter converted into glucuronide conjugates (156).

Enzymes involved in sulphation and glucuronidation are also found in the kidney, liver, and intestinal mucosa. In the intestine, curcuminoids, including curcumin, are conjugated to glucuronide, then form a glucuronide conjugates in the portal vein and subsequently conjugate into glucuronide/sulfate in the liver. Unlike oral administration, curcuminoids administrated intravenously in rats enter the blood circulation and dispose of in the liver as a free form (157).

To date, there are two known phases of curcumin metabolism, phase I and phase II. Phase I metabolism involves saturating double bonds to form several curcumin metabolites such as di, tetra, hexa, and octahydrocurcumin, whereas, in phase II metabolism, glucuronide or sulfate is conjugated to curcumin as well as its metabolites (154). Curcumin and its metabolites can form conjugation to any of their phenol-OH groups (21). Additionally, curcumin metabolism may occur in the intestine by microorganisms such as *Escherichia coli* whereby the NADPH-dependent enzyme converts curcumin into di- and tetrahydrocurcumin (158).

Curcumin degradation products including its metabolites may involve in the observed therapeutic effects. A few curcumin metabolites have been investigated in preclinical studies, for example, DHC exhibited lipid biosynthesis and increased lipid oxidation in HepG2 and LO_2_ cells. A molecular docking study revealed that DHC showed more affinity for binding to the phospholipase A2 active site than curcumin, indicating that DHC possesses anti-inflammatory activity (159), decreasing oxidative stress by upregulating NRF2, decreasing insulin resistance by increasing glucose uptake, and modulating the PI3K/AKT pathway (160).

Based on the chemical structure, THC is more stable and soluble in an aqueous medium than curcumin, as the former lacks α, β dienes, so it is unable to form Michael adducts with intracellular proteins. Nevertheless, the therapeutic activities of THC have been reported such as anti-aging, anti-neurodegeneration, and anticancer effects with significant antioxidant activity, so it can prevent human diseases related to oxidative stress (36). Hexahydrocurcumin (HHC), a hydrogenated metabolite of curcumin, is not well studied but it has been documented to have anticancer, antioxidant, antiinflammatory, and cardioprotective activities. Some of which have been reported to be more potent than the parent compound, curcumin, such as free radical scavenging activities (161). Octahydrocurcumin (OHC) exhibits several activities similar to the parent compound, curcumin, while its antitumor and anti-inflammatory activities were more potent than curcumin (162–164).

### Metabolism of Curcumin by Metabolizing Enzymes

Drug metabolism is categorized into phase I and phase II metabolism. Cytochrome P450 (CYP) enzymes, a large superfamily of heme proteins, play a major role in modulating phase I metabolism by catalyzing oxidative biotransformation of drugs and xenobiotics (165, 166). At least 57 CYP genes have been identified in humans. Of 18 families and 44 subfamilies human CYPs, only families 1–3 are most active in hepatic metabolism with five of them (CYP 1A2, 2C9, 2C19, 2D6, and 3A4) contributing to metabolism of 75% of drugs (165, 167). Drug therapy can induce or inhibit CYP enzymes resulting in drug-drug interaction. Induction or inhibition of CYP may decrease or increase plasma drug levels causing adverse drug reactions or toxicity (168). After CYP enzyme metabolism, most drugs are metabolized by conjugation reaction in phase II metabolism. Phase II enzymes contribute significantly to metabolic inactivation of drug compounds and biotransformation of xenobiotics to make them easier to excrete from the body. Phase II enzymes are mainly transferases, including glutathione S-transferases (GSTs), thiopurine-S-methyltransferase (TPMT), sulfotransferases (SULTs), uridine-diphosphate glucuronosyltransferases (UGTs), N-acetyltransferases (NATs), and NADH quinone oxidases (169).

Curcumin can modulate the activity of drug-metabolizing enzymes, both phase I and phase II metabolic enzymes (170). Curcumin is reported to significantly inhibit CYP2D6 activity in *in vitro* as well as *in vivo*. *In vitro* studies using human liver microsomes showed that 100 μg/mL of tumeric extract inhibited about 70% dextrorphan (metabolites of dextromethorphan mediated by CYP2D6) formation. Meanwhile, *in vivo* model exhibited a significant increase in human urinary metabolic ratio of dextromethorphan/dextrorphan after administration of tumeric extract (171). Similarly, CYP inhibitory activity assay using human recombinant CYP enzymes demonstrated that curcumin inhibited CYP2D6 by 60% at concentration of 120 μM and effectively inhibited CYP3A4 by almost eliminating the enzyme function at concentration ≥ 60 μM (172). In contrast, other *in vivo* and *ex-vivo* studies found that curcumin significantly activated CYP3A4. The study showed that 50 and 100 mg/Kg of curcumin markedly reduced the AUC_0−540_ of everolimus (a probe drug of P-glycoprotein/CYP3A4) by 70.6 and 71.5%, respectively (173). Meanwhile, Mach et al. reported that curcumin in liposomal formulation did not inhibit CYP2D6 and CYP3A4 activity, but liposomal curcumin at concentration of 58.3 μM inhibited CYP2C9 (10.5%) and CYP2C8 (22.5%) by *in vitro* assay (174).

Chen et al. investigated that 14 days of treatment with curcumin after caffeine administration significantly repressed CYP1A2 activity by 28.6% and significantly induced CYP2A6 activity by 48.9% in human subjects, but had no significant effect on N-acetyltransferase (NAT2) and xanthine oxidase (XO) (175). Volak et al. (176) reported that by *in vitro* assay using human liver microsomes, curcuminoid extract exhibits moderate to potent inhibitory activity (IC50<50 μM) toward various CYPs in the following ascending order CYP2C19 > CYP2B6 > CYP2C9 > CYP3A. In contrast, curcuminoid extract exhibits low inhibitory activity of CYP2D6, CYP1A2, and CYP2E1 (IC50 > 60 μM). Meanwhile, pure synthetic curcuminoids (curcumin, demethoxycurcumin, and bisdemethoxycurcumin) show similar inhibition to the CYP3A and CYP2C9 activities (176).

Several studies reported that curcumin modulates phase II metabolism enzymes. Curcuminoids can inhibit acetaminophen glucuronidation (by UGT enzyme) and acetaminophen sulfonation (by SULT enzyme) in LS180 cells. Curcumin is a more significant potent inhibitor of acetaminophen glucuronidation than demethoxycurcumin and bisdemethoxycurcumin, but compared to other curcuminoids, curcumin is the least potent inhibitor of acetaminophen sulfonation (176). In addition, curcumin is a potent inhibitor of phenol sulfotransferase (SULT1A1) in human liver, kidney, colon, duodenum, and lung (177). However, in a clinical study, short-term use (2 days) of curcuminoid/piperine combination is unlikely to affect significantly the pharmacokinetic disposition of drugs involving UGT and SULT (178).

In addition, curcumin was reported to modulate GST activity. Liu et al. (179) investigated the therapeutic potential of curcumin and resveratrol in mice bearing lung carcinoma induced by benzo(a)pyrene. The result showed that curcumin and a combination of curcumin-resveratrol treatment for 22 weeks significantly increased GST level by 14 and 18%, respectively, compared to benzo(a)pyrene treatment (179). Similarly, a study reported that 2 weeks of curcumin oral administration (400 mg/kg) in mice increased GST activity by 20% (180).

### Curcumin and the Intestinal Microbiome

Gut microbiota has recently gained great attention due to their role in understanding the pathobiology of diseases, efficacy of treatment, as well as diet and nutrition that affect human health (181). There is increasing evidence supporting the concept that the gut microbiota is responsible for the regulation of metabolic disorder including obesity, weight gain, and insulin sensitivity (162–164). Phyla Firmicutes and Bacteroidetes are associated with genetic obesity in rodents (182, 183), but efficacy may differ among specific genera and species. Bacteroides, for instance, are linked to a high fat/low-fiber intake, while Prevotella are associated with complex carbohydrate intake (184). In-depth knowledge of diet-microbiota interactions is also important to identify the risk factors of metabolic disorders closely related to diet and nutrition. The gut microbiome can be modified and regulated by probiotics and/or prebiotics, such as non-digestible carbohydrates (163, 185), that are considered as part of therapeutic strategies (186).

A preclinical study of obese mice showed that dietary supplementation of prebiotic oligofructose decreased *Streptococcus intermedius*, a cytolysin-producing microorganism that normally disturbs intestinal wall permeability. The human intestinal mucin-degrading bacterium, *Akkermansia muciniphila*, was also increased after the administration of prebiotics and correlated with elevated levels of Glucagon-like peptide-1 (GLP-1) and Glucagon-like peptide-2 (GLP-2) in L-cells (187). This organism is associated with metabolic-regulating proteins (188). In addition, the elevated concentration of these GLPs can improve gut barrier function and metabolism *via* the metabolism of fatty acids to short-chain fatty acids (189). Oral intake of curcumin enhanced the intestinal barrier function, improved glucose tolerance, and lowered circulating LPS levels in Western diet-fed LDL receptor knockout (LDLR) mice (190). Curcumin can act as a prebiotic to influence the microbiome, consequently, influence metabolic imbalance such as obesity (191). A double-blind, randomized, placebo-controlled pilot study revealed turmeric and curcumin alter gut microbiota, including the increases in most *Clostridium* spp., *Bacteroides* spp., *Citrobacter* spp., *Cronobacter* spp., *Enterobacter* spp., *Enterococcus* spp., *Klebsiella* spp., *Parabacteroides* spp., and *Pseudomonas* spp. and the reduction of several *Blautia* spp. and most *Ruminococcus* spp. (192). Although clinical trials are challenging, especially introducing prebiotics and probiotics to modulate the gut microbiome, constant and in-depth studies must be performed as there are several differences in metabolic profiles determined by gut microbiota (193). The microbiota response to treatment is highly personalized. The breakdown of curcumin and the biotransformed products must also be elucidated, as curcumin degradation products and/or metabolites possibly access the systemic circulation and metabolic system, albeit at great variability. Thus, the results from clinical trials must be facilitated by concomitant biofluids marker assessments to investigate curcumin administration and the correlated clinical outcomes.

## Conclusion and Future Perspectives

Preclinical findings of promising natural products frequently encounter challenges in clinical trials. Moreover, the diversity of clinical results is due to the divergence of metabolic phenotypes and physiological processes that affect disease progression in individuals/populations. Differing individual pharmacokinetic and dynamic profiles also affect the individual's response to natural products. Since MS is multifactorial, assessment of a single disease parameter after treatment is not sufficient, therefore large-scale studies and efforts are required to minimize bias. Some key points for further research and development include:

1. Oxidative stress and chronic inflammation are key factors in the pathogenesis of MS (194), with higher levels of oxidative stress markers observed in subjects with MS (195). Chronic hyperglycemia leads to oxidative stress leading to complications in patients with diabetes (194). In addition, fat accumulation in obese patients correlates with systemic oxidative stress in humans and mice, increased NADPH oxidase, and decreased endogenous antioxidant enzymes (196). Excess reactive oxygen species, chronic inflammation, increased fat accumulation, and insulin resistance leads to oxidative stress, whereby the antioxidant capacity of the human body is exceeded. Administration of curcumin, an antioxidant, sometimes failed to eliminate the excess free radicals, therefore an increased dose, delivery formulation, and duration of administration require further investigation. Elucidation of the effects of curcumin in healthy subjects before the excess oxidative stress occurs and can no longer be anticipated by single/mono phytochemical intervention should also be evaluated by measuring the ratio of pro-oxidants to antioxidants.

2. There is evidence that curcumin is degraded upon release into the physiological system or media regardless of the delivery mechanism. The combination of curcumin with various nutraceuticals may improve delivery, efficacy, and reduce toxicity, offering pleiotropic effects or modulation of multiple targets. Also, the use of crude turmeric extract might have more beneficial effects than the individual active compound, curcumin. When individual curcumin is explored, structural modification of curcumin is required to improve the bioavailability as well as the rate of metabolism. Investigations of the effects of curcumin in combination with other agents are ongoing, as well as a few clinical studies. Nonetheless, more, larger well-controlled clinical trials are necessary to evaluate the efficacy of each formulation compared to the parental compound focusing on specific disease targets.

3. The development of analogs and delivery systems are also ongoing to overcome the low bioavailability of curcumin, such as lipid vesicles, nanoparticles, and nanofibers. Besides, alternative routes of curcumin administration may enhance the therapeutic effect. One consideration that may require attention is the narrow therapeutic window leading to off-target toxicity.

4. Improved preclinical models are also required as animal model physiology is frequently different from human biology, accounting for the challenge in translating preclinical findings to the clinic. The stability and/or reactivity of a compound in assay buffer, media, and chemicals must be characterized for a rigorous and valid method. Also, the selection of the molecular target and pharmacology network should be identified, including the potency of off-target effects.

5. Degradation products or curcumin metabolites, including those produced by the gut microbiome, are of interest. Numerous curcumin studies have overlooked the investigation of the degradation products or curcumin metabolites, whether they share similar targets and responses in *in vivo* studies and clinical trials. There is much scope to investigate the role of gut microbiota on the pharmacological activity of curcumin regardless of the low systemic circulation.

6. Based on clinical studies and meta-analysis, curcumin/curcuminoid/turmeric might effectively lower fasting plasma glucose and HbAic, triglyceride, total cholesterol, and LDL, but no effect on patients with no diabetic history. The compound(s) show no effect on improving SBP and HDL-C. Metabolomics and pharmacometabolomics studies are particularly necessary to advance the precise and personalized utilization of curcumin, particularly in the case of MS. Metabolic profiling should be employed synergistically with genomic analyses (i.e. genes that encode metabolizing enzymes and drug transporters), as employing genetic information only (pharmacogenomics) may not lead to comprehensive disease phenotyping and translate into a therapeutic response.

## Author Contributions

AN reviewed, drafted and designed, and conceived the manuscript. FC, MPW, and MM reviewed and drafted the manuscript. MW revised the manuscript. MS revised, designed, and conceived the manuscript. All authors contributed to the article and approved the submitted version.

## Funding

APC was paid by UM6P.

## Conflict of Interest

The authors declare that the research was conducted in the absence of any commercial or financial relationships that could be construed as a potential conflict of interest.

## Publisher's Note

All claims expressed in this article are solely those of the authors and do not necessarily represent those of their affiliated organizations, or those of the publisher, the editors and the reviewers. Any product that may be evaluated in this article, or claim that may be made by its manufacturer, is not guaranteed or endorsed by the publisher.
